# Supplementary material for: Identification of photoperiod-induced specific miRNAs in the adrenal glands of Sunite sheep (Ovis aries)
Source: Front Vet Sci. 2022 Jul 22;9:888207. doi: 10.3389/fvets.2022.888207 (PMC9354845; doi:10.3389/fvets.2022.888207)
Supplement: Supplementary file 1 [file Data_Sheet_1.ZIP › Supplementary file/miRDeep2 file.zip!/miRDeep2 file/Novel_Structure/SPLP42A2/Novel_540.pdf]

Provisional ID	:	NC_019467.2_21511
Score total	:	16.8
Score for star read(s)	:	3.9
Score for read counts	:	9.3
Score for mfe	:	2
Score for randfold	:	1.6
Score for cons. seed	:	
Total read count	:	30
Mature read count	:	29
Loop read count	:	0
Star read count	:	1

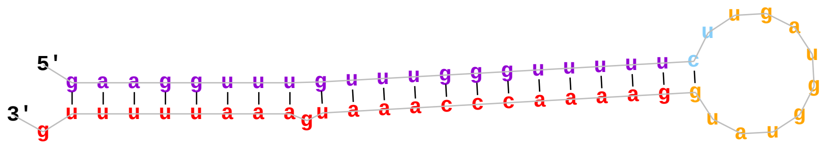

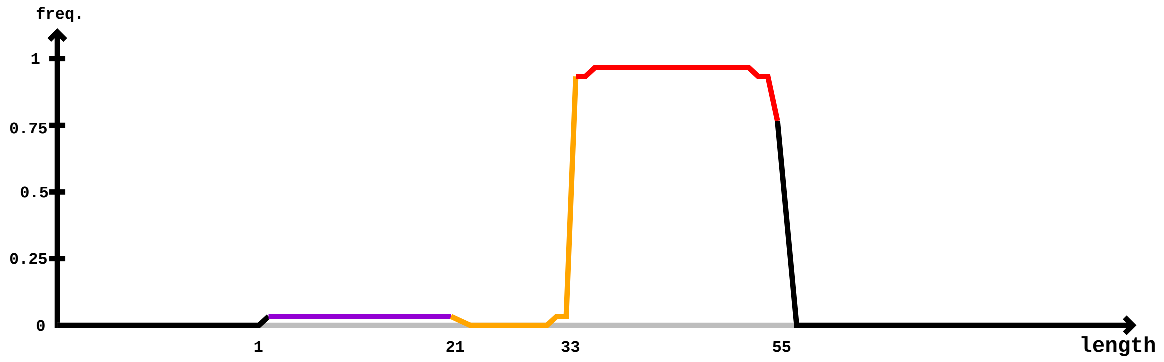

Star **Mature**

```
5'- guaaauuauuggguuggccagaagguuuguuuggguuuuucuugaugguauggaaaacccaaaugaaauuuuuggccaaccuaauacuuaauuucucagucuguucauuc   -3'    obs
    guaaauuauuggguuggccagaagguuuguuuggguuuuucuugaugguauggaaaacccaaaugaaauuuuuggccaaccuaauacuuaauuucucagucuguucauuc          exp
    ......((((((((((((((((((.((((((((((((((((..........)))))))))))))))).))))))))))))))))))........................   reads     mm        sample
    ....................gaagguuuguuuggguuuuu......................................................................   1         0         seq
    ..................................................uggaaaacccaaaugaaCuuu.......................................   1         1         seq
    ....................................................gaaaacccaaaugaaCuuuuu.....................................   5         1         seq
    ....................................................gaaaacccaaaugaaCuuuuug....................................   21        1         seq
    ....................................................gaaaacccaaaugaaCuuuuugg...................................   1         1         seq
    ......................................................aaacccGaaugaaauuuuug....................................   1         1         seq
```
